# *Nannochloropsis oceanica* as a Microalgal Food Intervention in Diet-Induced Metabolic Syndrome in Rats

**DOI:** 10.3390/nu13113991

**Published:** 2021-11-09

**Authors:** Ryan du Preez, Marwan E. Majzoub, Torsten Thomas, Sunil K. Panchal, Lindsay Brown

**Affiliations:** 1 Functional Foods Research Group, University of Southern Queensland, Toowoomba, QLD 4350, Australia; r.dupreez@cqu.edu.au (R.d.P.); S.Panchal@westernsydney.edu.au (S.K.P.); 2 Centre for Marine Science and Innovation, School of Biological, Earth and Environmental Sciences, University of New South Wales, Sydney, NSW 2052, Australia; m.majzoub@unsw.edu.au (M.E.M.); t.thomas@unsw.edu.au (T.T.)

**Keywords:** *Nannochloropsis oceanica*, microalgae, metabolic syndrome, gut microbiota, eicosapentaenoic acid

## Abstract

The microalgal genus *Nannochloropsis* has broad applicability to produce biofuels, animal feed supplements and other value-added products including proteins, carotenoids and lipids. This study investigated a potential role of *N. oceanica* in the reversal of metabolic syndrome. Male Wistar rats (*n* = 48) were divided into four groups in a 16-week protocol. Two groups were fed either corn starch or high-carbohydrate, high-fat diets (C and H, respectively) for the full 16 weeks. The other two groups received C and H diets for eight weeks and then received 5% freeze-dried *N. oceanica* in these diets for the final eight weeks (CN and HN, respectively) of the protocol. The H diet was high in fructose and sucrose, together with increased saturated and *trans* fats. H rats developed obesity, hypertension, dyslipidaemia, fatty liver disease and left ventricular fibrosis. *N. oceanica* increased lean mass in CN and HN rats, possibly due to the increased protein intake, and decreased fat mass in HN rats. Intervention with *N. oceanica* did not change cardiovascular, liver and metabolic parameters or gut structure. The relative abundance of Oxyphotobacteria in the gut microbiota was increased. *N. oceanica* may be an effective functional food against metabolic syndrome as a sustainable protein source.

## 1. Introduction

Microalgae are unicellular organisms that, in the presence of sunlight, convert carbon dioxide into biomass [1]. The members of the microalgal genus *Nannochloropsis* can be defined as biorefineries to produce biofuels, animal feed supplements and pigments [2,3]. *Nannochloropsis* species have a wide range of applications in biotechnology, using techniques to modify biomass composition [4]. *Nannochloropsis* species contain 28.7–40.4% carbohydrates, 22.2–37.4% crude protein and 15.1–21.7% total lipids on dry weight basis [5] as well as minerals, vitamins and antioxidants such as carotenoids [5,6]. *Nannochloropsis* species contain polyunsaturated fatty acids (PUFA), mainly eicosapentaenoic acid (EPA), polyphenols, carotenoids and vitamins with toxicological tests on animals confirming the safety of this microalga for consumption in food [7]. There are six known species of *Nannochloropsis*, including *N. oceanica*, *N. gaditana* and *N. oculata*. Baseline information on the biology of *N. oceanica* has been published for use in the risk analysis of genetically modified *N. oceanica* in Australia [8]. As one example of its potential, *N. oceanica* strains isolated in Southeast Queensland, Australia, showed improved lipid characteristics [9].

Edible microalgae may have therapeutic potential such as the reduction of signs of metabolic syndrome including hypertension, obesity, fatty liver and systemic inflammation [10]. *Nannochloropsis* has been proposed as an appropriate nutritional supplement to increase PUFA and EPA intake as its EPA content can be rapidly increased, for example by low-level UV-C radiation [3,7]. Its introduction in the human diet is eco-sustainable and can replace products currently sourced from fish capture [7,11,12]. Few studies have tested *Nannochloropsis* species on metabolic syndrome. Species such as *N. gaditana* and *N. oculata* alleviated oxidative stress and inflammation in streptozotocin-induced diabetic rats [13], and a protein hydrolysate of *N. oculata* showed antihypertensive effects [14]. Omega-3 fatty acids including EPA decreased the signs of metabolic syndrome in the same rat model of diet-induced metabolic syndrome used in the current study [15]. However, there are no similar studies on *N. oceanica*, a source of EPA, on the combination of pathological changes that define metabolic syndrome.

This project has tested an Australian strain of *N. oceanica* farmed locally in a covered pond under controlled conditions [9]. The aim of this study was to determine whether freeze-dried *N. oceanica* could reverse the signs of diet-induced metabolic syndrome. We tested the microalga using a validated diet-induced model of metabolic syndrome in rats that closely mimics the symptoms of human metabolic syndrome [16]. Cardiovascular, liver and metabolic health parameters were defined after 8 weeks’ dietary intervention, with *N. oceanica* starting 8 weeks after initiation of the obesogenic diet. As the microalgal cells are difficult to lyse in vivo to release EPA, we used the whole biomass to test the effect of components other than EPA, which has already been tested in metabolic syndrome [15].

We measured cardiovascular parameters including systolic blood pressure, left ventricular diastolic stiffness, cardiac inflammatory cells and collagen deposition in the heart; liver parameters including plasma liver enzyme activities, inflammatory cells and fat vacuoles in the liver; and metabolic parameters including body weight, total cholesterol and triglyceride concentrations and glucose and insulin tolerance tests. Further, as functional foods may reverse obesity-induced changes in the gut microbiota [17,18], we characterised the changes in its composition after microalgal treatment. We hypothesised that 5% *N. oceanica* supplementation for the last eight weeks of the protocol will reverse the changes induced by the high-carbohydrate, high-fat diet. The mechanisms of these hypothesised effects could include the provision of essential amino acids to provide an increased lean mass as well as the actions of carotenoids, chlorophyll a and the omega-3 fatty acid EPA to decrease infiltration of inflammatory cells into organs such as the heart and liver.

## 2. Materials and Methods

### 2.1. *Nannochloropsis oceanica* Source

*N. oceanica* (strain CS-179) was cultivated and harvested by Teraform microalgae farm, Miles, QLD, Australia during June and July 2018. The harvested paste (40 L) was transported frozen to the University of Southern Queensland, Toowoomba, QLD, Australia in sealed plastic containers. The paste was kept frozen until it was freeze-dried (Martin Christ Alpha 2–4 LD plus, John Morris Scientific, Murarrie, QLD, Australia). Batches of 5 L were freeze-dried for 36–48 h at 0.011 mbar and −60 °C. The powder was stored at 4°C in sealed plastic containers until feeding or analysis. The composition of *N. oceanica* was obtained as described previously [19].

### 2.2. Rats and Diets

Male Wistar rats (8–9 weeks old; 336 ± 2 g, *n* = 48) were sourced from Animal Resource Centre, Murdoch, WA, Australia and housed at the University of Southern Queensland Animal Facility before being randomly divided into four groups (12 rats per group). Two groups received either corn starch (C) or high-carbohydrate, high-fat diets (H) for the full 16 weeks [16]. The other two groups (CN and HN) received C or H diets for the first eight weeks and then received C or H diets with 5% freeze-dried *N. oceanica* for the final eight weeks. The composition of C and H diets is described in our previous study [16]. H and HN rats were given fructose in drinking water (25% *w*/*v*) along with H diet.

### 2.3. Rat Measurements

Rats were anaesthetised using isoflurane for measurements of body composition using dual-energy X-ray absorptiometry (DXA), systolic blood pressure and abdominal circumference measurement [20]. DXA measurements were performed on rats during week 16 of the protocol using a Norland XR46 DXA instrument (Norland Corp., Fort Atkinson, WI, USA) [16]. Systolic blood pressure was measured at 8 and 16 weeks under anaesthesia using an MLT1010 Piezo-Electric Pulse Transducer (ADInstruments, Bella Vista, NSW, Australia) and an inflatable tail-cuff connected to an MLT844 Physiological Pressure Transducer (ADInstruments) connected to a PowerLab data acquisition unit (ADInstruments) [16].

For oral glucose tolerance tests, all rats were food-deprived overnight (~12 h). H and HN rats were given normal tap water without fructose during the food deprivation period. After overnight food deprivation, basal blood glucose concentrations were measured in blood collected from the tail vein and analysed using glucometer (Freestyle lite, Abbott Diabetes Care, VIC, Australia). The rats were then gavaged with 2 g/kg aqueous glucose solution, and blood glucose measurements were performed at 30, 60, 90 and 120 min after glucose loading using tail vein prick method [16]. For insulin tolerance tests, all rats were deprived of food and fructose water for 4–5 h. Blood glucose concentrations were then measured at the end of this food deprivation period. Following this, the rats were intraperitoneally injected with 0.75 IU/kg body weight insulin-R (Eli Lilly, West Ryde, NSW, Australia). Tail vein blood samples were analysed for blood glucose concentrations at 30, 60, 90 and 120 min following insulin administration. If the blood glucose concentration dropped below 1.1 mmol/L, rats were removed from the test and immediately given 4 g/kg body weight glucose solution by oral gavage to reverse hypoglycaemia [15].

Following euthanasia with intraperitoneal Lethabarb (pentobarbitone sodium, 100 mg/kg; Virbac, Peakhurst, NSW, Australia), heparin (~200 IU) was injected into right femoral vein before blood collection, centrifugation and plasma isolation. Following blood collection, hearts were removed to measure diastolic stillness using isolated Langendorff heart preparation [16]. Organ weights were collected for right and left ventricles, liver and retroperitoneal, epididymal and omental fat shortly after euthanasia. These weights were normalised relative to the tibial length at the time of their removal (in mg/mm) [16]. Organs were also collected for histological analyses and processed as previously described [16]. The liver sample was taken from the largest lobe close to the hepatic portal vein.

After euthanasia, two or three faecal pellets were collected from the colon of each rat and processed as described previously to obtain the gut microbiota composition [20,21]. Gut microbiota diversity profiling was performed based on 16S rRNA gene sequencing. Bacterial communities from faecal samples were investigated by sequencing 16S rRNA gene amplicons. 341F and 785R primers were used to amplify the V_3_-V_4_ regions of the 16S rRNA gene. Data were presented and analysed for statistical significance as detailed in previous studies [20,21].

## 3. Results

### 3.1. *Nannochloropsis oceanica*

**Figure 1:**
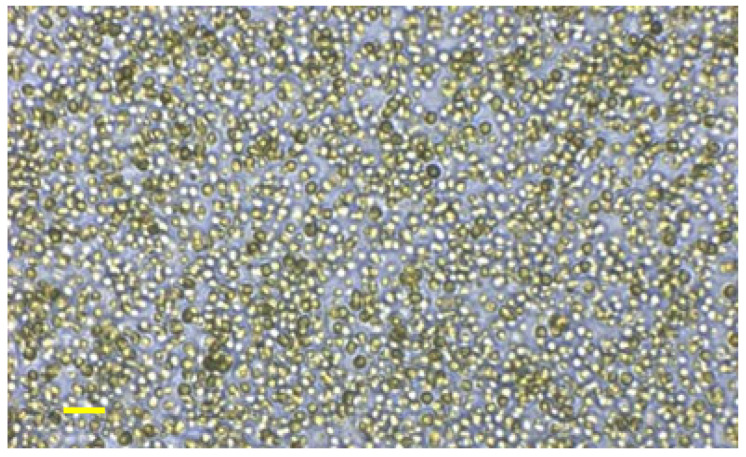
Intact cell wall of *Nannochloropsis oceanica* under brightfield microscopy. Scale bar is 10 µm (10×).

The cell wall of *N. oceanica* was intact (Figure 1). The *N. oceanica* biomass showed high protein content including essential amino acids together with high PUFA content, predominantly EPA, as well as vitamins and carotenoids (Table 1). The energy content was 1571 kJ/100 g algal powder.

### 3.2. Physiological Variables

As expected, the body weight of H rats was higher than C rats (Table 2). The body weight of HN rats was not different from H rats, while the CN rats were heavier than C rats. Lean mass was not different between C and H rats. For CN and HN rats, the lean mass was higher than their respective controls. Bone mineral content was higher in H and HN rats compared to C and CN rats. Bone mineral density of H rats was higher than that of C rats. The bone mineral density of CN and HN rats was not different from the respective controls. Food intake was higher in C rats compared to H rats. CN rats had lower food intake than C rats. HN rats had similar food intake to H rats. Water intake was higher in H rats compared to C rats and further increased in CN and HN rats, but this increase with *N. oceanica* intervention was not associated with an increased energy intake; these changes may be caused by the increased salt or protein intake. Energy intake was highest in H rats compared to C rats. HN rats had similar energy intake as H rats. CN rats had the lowest energy intake (Table 2).

Whole body fat mass by DXA was higher in H rats compared to C rats. CN rats had similar whole-body fat mass as C rats, but HN rats had lower fat mass than H rats. Total abdominal fat was higher in H rats compared to C rats, and HN rats had less abdominal fat than H rats. Epididymal and omental fat pads were not different from their respective controls. Retroperitoneal fat was higher in H rats compared to C rats, while HN rats had less retroperitoneal fat compared to H rats. Values in CN rats were not different from C rats (Table 2).

Plasma triglyceride concentrations were higher in H rats compared to C rats, whereas HN rats were similar to H rats, while CN rats were higher than C rats. Plasma non-esterified fatty acids were the same for C and H rats, but CN and HN rats were higher than their respective controls. Plasma total cholesterol concentrations were unchanged among all groups (Table 2). H rats had higher 120-min blood glucose concentrations and area under the curve compared to C rats. CN and HN rats were not different from their respective controls. H rats had higher 120-min blood glucose concentrations and area under the curve after insulin administration compared to C rats; CN rats were higher than C rats; and HN rats were higher than H rats (Table 2).

After eight weeks, systolic blood pressures of H and HN rats were higher than of C and CN rats. Systolic blood pressures in H rats were higher at 16 weeks than in C rats. CN and HN rats were not different from their respective controls. Left ventricular diastolic stiffness was higher in H rats compared to C rats. CN and HN rats were different from their respective controls. Left ventricular weights with septum and right ventricular wet weights were unchanged in all groups.

**Figure 2:**
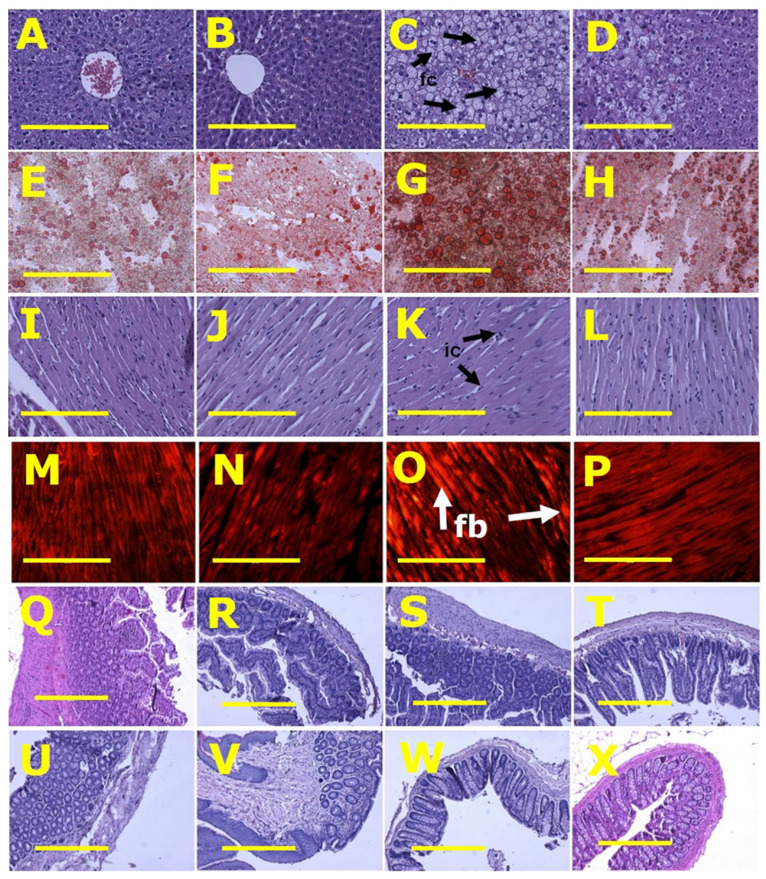
Histological analysis of liver, heart, ileum and colon. (**A**–**D**) showing haematoxylin and eosin staining; (**E**–**H**) showing oil red O staining to identify liver fat deposition; (**I**–**L**) showing haematoxylin and eosin staining to identify heart inflammation; (**M**–**P**) showing picrosirius red staining to identify myocardial collagen deposition; (**Q**–**T**) showing haematoxylin and eosin staining of ileum; and (**U**–**X**) showing haematoxylin and eosin staining of colon in rats fed with corn starch diet (**A**,**E**,**I**,**M**,**Q**,**U**), rats fed with corn starch diet + *Nannochloropsis oceanica* (**B**,**F**,**J**,**N**,**R**,**V**), rats fed with high-carbohydrate, high-fat diet (**C**,**G**,**K**,**O**,**S**,**W**) and rats fed with high-carbohydrate, high-fat diet + *Nannochloropsis oceanica* (**D**,**H**,**L**,**P**,**T**,**X**). Fat cells = fc; inflammatory cells = ic; collagen = fb. Scale bar is 200 µm for (**A**–**P**) (20×) and 100 µm for (**Q**–**X**) (10×).

Left ventricles from H rats showed increased infiltration of inflammatory cells and collagen deposition whereas these changes were not seen in left ventricles from C rats. CN and HN rats were not different from their respective controls (Figure 2). Livers from H rats showed increased fat vacuole size and infiltration of inflammatory cells compared to livers from C rats, while HN rats had decreased fat vacuole size and fewer inflammatory cells compared to H rats (Figure 2). Plasma activities of alanine transaminase and aspartate transaminase were not different between all groups (Table 2).

### 3.3. Gut Structure and Microbiota

Histology of ileum and colon did not show any structural abnormalities in the ex-perimental groups demonstrated by normal crypt depth, villi length, goblet cells and lack of inflammatory cell infiltration (Figure 2).

**Figure 3:**
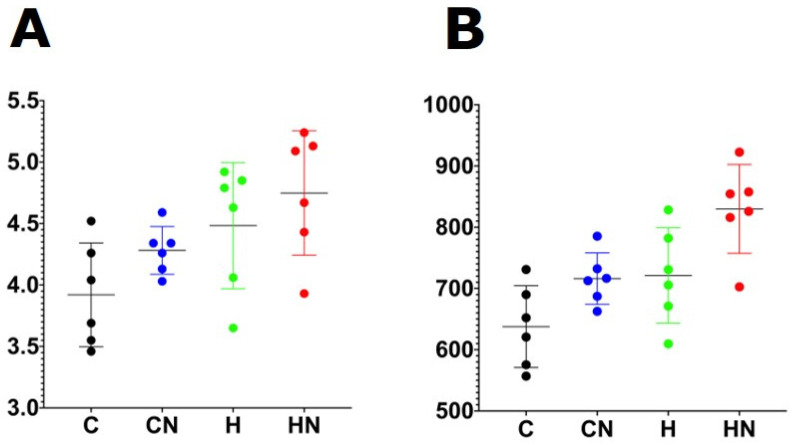
(**A**) Shannon diversity and (**B**) richness of faecal samples. C, rats fed with corn starch diet; CN, rats fed with corn starch diet + *Nannochloropsis oceanica*; H, rats fed with high-carbohydrate, high-fat diet; HN, rats fed with high-carbohydrate, high-fat diet + *Nannochloropsis oceanica*.

**Figure 4:**
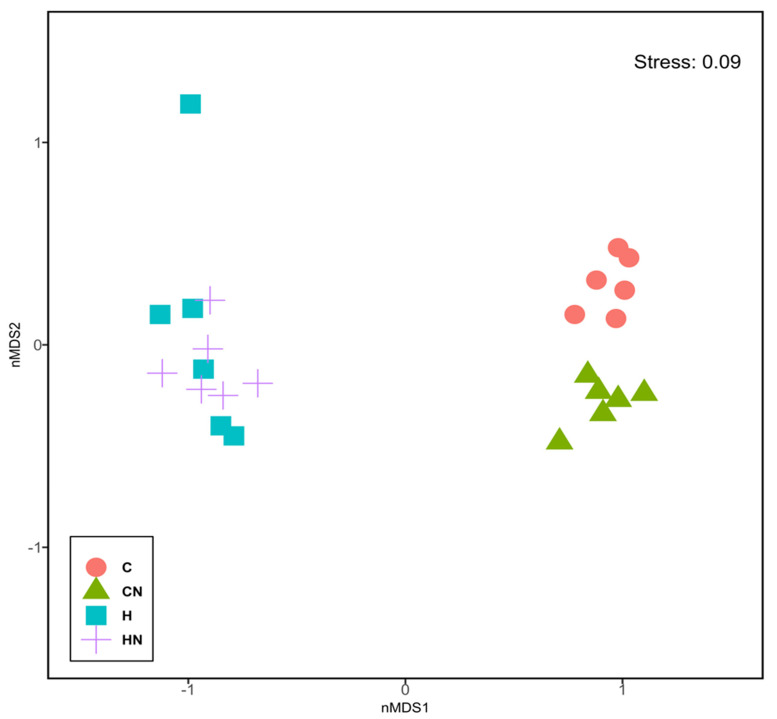
Non-metric multi-dimensional scaling plot of bacterial gut communities. C, rats fed with corn starch diet; CN, rats fed with corn starch diet + *Nannochloropsis oceanica*; H, rats fed with high-carbohydrate, high-fat diet; HN, rats fed with high-carbohydrate, high-fat diet + *Nannochloropsis oceanica*.

For gut microbiota characterisation, a total of 788,078 quality-filtered sequences were clustered into 1282 zOTUs; Good’s coverage score of 99.69 ± 0.08% suggested an almost full recovery of bacterial communities. Shannon’s diversity and richness indices were unchanged among the groups (Figure 3). Diet and *N. oceanica* affected the overall bacterial community structure individually as well through their interaction (Figure 4, Supplementary Tables S1–S6).

**Figure 5:**
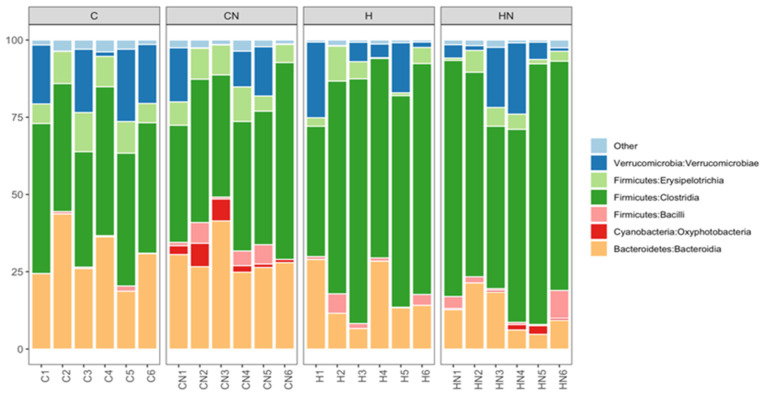
Taxonomic profiles of bacterial communities of all faecal samples shown at the class level. C, rats fed with corn starch diet; CN, rats fed with corn starch diet + *Nannochloropsis oceanica*; H, rats fed with high-carbohydrate, high-fat diet; HN, rats fed with high-carbohydrate, high-fat diet + *Nannochloropsis oceanica*.

Bacteroidia, Oxyphotobacteria, Bacilli, Clostridia, Erysipelotrichia and Verrucomicrobiae were the most abundant bacterial classes in the faecal samples (Figure 5). Actinobacteria, Coriobacteriia, Melainabacteria, Deferribacteres, Saccharimonadia, Planctomycetacia, Alphaproteobacteria, Deltaproteobacteria, Gammaproteobacteria and Mollicutes were observed at lower abundance levels (<1%) in some faecal samples.

The relative abundance of bacteria from the class Bacteroidia and Erysipelotrichia was increased in C and CN rats (Bacteroidia: C, 29.00%; CN, 29.63%; *p* > 0.05; Erysipelotrichia: C, 9.31%; CN, 8.21%; *p* < 0.01) compared to H and HN rats (Bacteroidia: H, 17.17%; HN, 12.12%; *p* > 0.05; Erysipelotrichia: H, 4.35%; HN, 3.93%; *p* < 0.01). An increase in the relative abundance of bacteria from the class Clostridia was observed in H and HN rats (H, 66.32%; *p* < 0.01; HN, 69.36%; *p* < 0.0001) compared to C and CN rats (C, 43.45%; CN, 45.46%). An increase in the relative abundance of bacteria from the class Oxyphotobacteria was observed in CN and HN rats (CN, 3.61%; HN, 1.02%; *p* > 0.05) compared to C and H rats (C, 0%; H, 0%) (Figure 5). A decrease in the relative abundance of bacteria from the class Bacilli was observed in HN rats (2.77%; *p* > 0.05) compared to CN rats (3.26%), while an increase was observed in H rats (2.27%; *p* > 0.05) compared to C rats (0.05%) (Figure 5). Similarly, the relative abundance of bacteria from the class Verrucomicrobiae was higher in C rats (13.93%; *p* > 0.05) compared to H rats (8.89%) and lower in CN rats (7.58%; *p* > 0.05) compared to HN rats (9.18%) (Figure 5).

The effects of diet and *N. oceanica* on the ratio of Firmicutes and Bacteroidetes (Supplementary Figure S1) bacterial communities at the family level (Supplementary Figure S2) and bacterial communities at the genus level (Supplementary Figure S3) are provided in the supplementary file. Detailed correlation analysis of gut microbiota with physiological parameters showed relationships between 12 physiological variables and gut microbiota in Supplementary Tables S6 and S7. The physiological variables most often related to changes in the gut microbiota were systolic blood pressure, liver wet weight and abdominal (retroperitoneal, epididymal and omental) fat pads (Table S6).

## 4. Discussion

The diet-induced changes in metabolic, cardiovascular and liver parameters in the rat model used in this project mimic the changes in human metabolic syndrome [16]. Interventions with seaweeds have been previously shown to reverse these changes [19,20,21]. This study shows that high-carbohydrate, high-fat diet-fed rats supplemented with the microalgae *N. oceanica* had higher lean mass and lower abdominal and liver fat than rats fed only the obesogenic diet. Further, the abundance of Oxyphotobacteria in the colon was changed. However, intervention with *N. oceanica* did not change cardiovascular parameters, lipid profile or glucose responses.

Microalgae are considered part of a healthy diet as they contain fatty acids, proteins, amino acids, pigments, vitamins and minerals [22]. Microalgae are a sustainable source of these compounds because they grow in a wide range of environments such as fresh, brackish and saline waters [23] and they do not compete with arable land or biodiverse landscapes [24]. Microalgal constituents are versatile and have potential applications in energy, pharmaceutical, cosmetics and food industries [25]. *Nannochloropsis* components such as whole biomass, pigments, long-chain PUFA, triglycerides, alkanes and alkenes have many biotechnological applications including production of biofuels [26], aquaculture, fish food, livestock feeds and wastewater treatment [27]. Because of these applications, *Nannochloropsis* grown for other uses could be diverted for the development of functional food products at minimal additional cost.

*Nannochloropsis* is nutritionally safe and can be used as a human health supplement [28]. Microalgae-supplemented food such as bread would address the general deficiency of omega-3 fatty acids and minerals, such as zinc, in the human population [2], although the change in colour may decrease consumer acceptance. *Nannochloropsis* can be added to food, such as bread [29] and pasta [30], to create highly nutritious functional foods. The addition of *N. gaditana* to bread changed the colour to green-yellow crust and crumb, suggesting an increased browning. The textural parameters of the bread such as hardness, chewiness and resilience were unchanged [29] whereas the appearance of pasta was minimally impacted with 10% replacement of wheat flour [30].

No studies have reported the effects of *N. oceanica* on changes in all components of metabolic syndrome using a single model, as in the current study. In rats, streptozotocin was used to produce acute pancreatic β-cell damage and induce hyperglycaemia [14]. Diabetic rats received *N. oculata* (10 and 20 mg/kg) for three weeks. *N. oculata* reduced serum concentrations of glucose, cholesterol, triglycerides and LDL and increased the serum concentrations of insulin and HDL-cholesterol. In another streptozotocin study, rats were fed with *N. gaditana* (10%) for two months [13]. *N. gaditana* supplementation decreased concentrations of glucose and HbA_1c_ and improved renal and hepatic functions while attenuating the oxidative stress and inflammation in diabetic rats. The marine-water microalga *N. oculata* and its extract minimised the pancreatic tissue damage and maintained the integrity of the genomic DNA [31]. *N. oculata* is a good source of omega-3 fatty acids, specifically EPA. Intervention with *N. oculata* suspension (10^8^ viable cells/animal) for 14 days had no effect on body weight, which is similar to the current study [32]. Using the same model of metabolic syndrome as the current study, ALA, EPA and DHA [15] improved cardiovascular and hepatic parameters. However, the EPA dose in this previous study was ~1300 mg/kg/day for 8 weeks, about five times higher than the EPA dose in the current study of around 260 mg/kg/day, also for 8 weeks. Further, the major four xanthophyll carotenoids in *N. oceanica* were present at 1940 mg/kg of the microalgal biomass which then gives a dose of approximately 4 mg/kg/day when mixed in the food. For comparison, a much higher dose of astaxanthin (200 mg/kg/day) given to Spontaneously Hypertensive Rats for 11 weeks reduced blood pressure [33]. These comparisons suggest that neither EPA nor the xanthophyll carotenoids are the major bioactive components of *N. oceanica* algal biomass. Further, this study tested *N. oceanica* without disrupting the cell structure, which is likely to further reduce the bioavailability of these components as ball-mill disruption enabled the protein and fatty acids to become bioavailable to mice [34]. It can be expected that the *N. oceanica* biomass, when used after processes such as ball milling [34], may improve the bioavailability of carotenoids and omega-3 fatty acids from the cell walls.

The gut microbiota plays an important role in health and disease [35]. Dietary interventions such as macro- and micro-algae can directly interact with the gut microbiota, leading to changes in physiological variables [35]. The search for microbial signatures of disease has led to the use of changes in the Firmicutes/Bacteroidetes ratio as a marker of obesity; however, use of this ratio may not be valid to determine health status because of lifestyle-associated variations in patients from a single population [36]. Our previous studies have shown interaction of polysaccharides from macroalgal interventions with the gut microbiota in improving metabolic and cardiovascular health [19,20,21]. As an example, our study on the macroalgae *Caulerpa lentillifera* showed correlations between gut microbiota and 15 physiological variables, especially oral glucose tolerance, liver weight and abdominal fat pads [21]. The current study extends this correlation to intervention with microalgae, suggesting that changes in gut microbiota are widely relevant in metabolic syndrome. Further, we have identified changes in Oxyphotobacteria with *N. oceanica* intervention.

Marine fatty fish such as salmon, mullet and mackerel are the main sources of EPA and DHA for human consumption [37]. However, due to the excessive and sometimes poorly regulated fishing industry, the depletion of worldwide fish stocks is straining the sustainability of production of omega-3 long-chain PUFA [38]. In contrast, microalga can be used for sustainable production of omega-3 PUFA [39] and so can be an important PUFA source for farmed fish [11,12]. Microalgae grow well in South-East Queensland, Australia [23]; hence, this may be a key location to provide good quality microalgae for Australian and international use. The biomass from *Nannochloropsis* species also contains high-value products such as other fatty acids, sterols and carotenoids with applications in food, cosmetic and pharmaceutical industries [40]. Defatted *Nannochloropsis* biomass is a good source of protein and carbohydrates which may have health benefits in addition to the increased EPA and carotenoids if the cell wall is broken [8]. Further, the dietary fibre from microalgal biomass could act as prebiotics to alter the gut microbiota leading to health benefits including reduced blood pressure, blood glucose, cholesterol, plasma triglycerides and LDL-cholesterol [41].

An advantage of this study was that the cell wall was not disrupted, and therefore, the effectiveness of microalgal components other than cell wall-bound EPA and carotenoids could be determined. A key feature of microalgae is the rigidity of the cell wall, which can limit the bioavailability of nutrients; hence, other studies have used several cell disruption methods such as mechanical, physical, chemical and enzymatic approaches [42] or solvent extraction [43]. Cell wall thickness in *Nannochloropsis* species varies from 63 to 119 nm due to the distinct genetic traits in each strain, with *N. oceanica* having one of the thickest cell walls [44]. Cell membrane disruption of *N. oceanica* may be necessary for optimal biological activity [45]. Health products including omega-3 fatty acids and vitamin D supplements can be obtained from *Nannochloropsis* using microwave, super-critical, ultrasound and enzyme-assisted extractions at industrial scales [46]. Our study shows that the biological activity of the biomass does not rely solely on EPA and carotenoids.

There may be a role for microalgal protein in providing a sustainable source of protein to augment diets that maintain weight loss. Typically, people regain weight after weight loss, with only diets with increased protein content having a beneficial effect in maintaining the reduced weight [47]. Animal protein consumption has been linked to abdominal adiposity and was generally detrimental to overall health in an adolescent population [48], whereas plant protein consumption was linked to better health. Therefore, microalgal protein may be a suitable alternative to animal proteins in maintaining a reduced body weight and health. Microalgal interventions may be useful as additives with other functional foods to increase the therapeutic effectiveness in metabolic syndrome.

The dose of 5% of diet in rats corresponds to approximately 30 g per day intake in adult humans [49]. This is a realistic and commercially viable dose in humans. Any higher doses may make it unrealistic and non-commercial, apart from decreasing the compliance and affordability.

## 5. Conclusions

*N. oceanica* intervention increased lean mass in rats, possibly due to the increased protein intake and decreased fat mass in obese rats, but this intervention did not change cardiovascular, liver and metabolic parameters or gut structure. As *N. oceanica* biomass can be produced sustainably in large quantities, it could be a source of essential amino acids and prebiotics that may improve health in chronic diseases such as metabolic syndrome. These are additional effects to the production of EPA and carotenoids by *Nannochloropsis* as a biorefinery. Further, the industrial usefulness of *Nannochloropsis* biomass for biofuels and animal feed supplements means that production of these amino acids and prebiotics can be undertaken using existing processes.

## Figures and Tables

**Table 1:** Nutrient composition of *Nannochloropsis oceanica* algae powder.

Component	Amount
Macronutrients (g/100 g)	
Protein	51.3
Essential amino acids (g/100 g)	
Lysine	3.5
Methionine	1.1
Isoleucine	2.1
Leucine	4.7
Threonine	2.6
Tryptophan	0.67
Valine	2.9
Arginine	2.9
Histidine	0.96
Phenylalanine	2.6
Lipids, moisture, carbohydrates (g/100 g)	
Fat	16.3
Saturated	3.1
Monounsaturated	4.9
Polyunsaturated	7.7
EPA	6
Omega-6	1.7
*Trans*	<0.01
Moisture	4
Total sugar	<0.01
Total fibre	10
Vitamins (mg/100 g)	
Vitamin A	0.025
Vitamin B1 (thiamine)	7
Vitamin B2 (riboflavin)	6.2
Vitamin B12 (cyanocobalamin)	0.17
Vitamin C (ascorbic acid)	320
Vitamin D	0.045
Vitamin E	35
Vitamin K	0.017
Carotenoids (mg/100 g)	
β-carotene	23.6
Lutein	248
Violaxanthin	146
Zeaxanthin	110

**Table 2:** Responses to *Nannochloropsis oceanica*.

Variables	C	CN	H	HN	*p* Value		
					Diet	Treatment	Interaction
Physiological variables							
0-week body weight, g	337 ± 1	336 ± 1	339 ± 1	337 ± 1	0.19	0.19	0.66
8-week body weight, g	366 ± 7 ^b^	386 ± 2 ^b^	445 ± 10 ^a^	430 ± 5 ^a^	<0.0001	0.66	0.004
16-week body weight, g	388 ± 10 ^c^	453 ± 6 ^b^	547 ± 14 ^a^	528 ± 10 ^a^	<0.0001	0.032	0.0003
16-week lean mass, g	292 ± 15 ^b^	328 ± 7 ^a^	299 ± 13 ^b^	323 ± 6 ^a^	0.92	0.003	0.53
16-week fat mass, g	75 ± 15 ^c^	91 ± 12 ^c^	230 ± 35 ^a^	172 ± 13 ^b^	<0.0001	0.26	0.05
8-week lean/fat mass proportion	6.2 ± 1.6 ^a^	5.8 ± 0.4 ^a^	2.1 ± 0.4 ^b^	2.8 ± 0.3 ^b^	<0.0001	0.82	0.42
16-week lean/fat mass proportion	3.8 ± 1.0 ^a^	4.3 ± 0.5 ^a^	1.5 ± 0.3 ^b^	2.0 ± 0.2 ^b^	0.0001	0.35	1.00
16-week bone mineral content, g	11.6 ± 0.3 ^b^	12.3 ± 0.4 ^b^	16.6 ± 1.1 ^a^	15.0 ± 0.5 ^a^	<0.0001	0.46	0.07
16-week bone mineral density, g/cm^2^	0.170 ± 0.003	0.166 ± 0.002	0.181 ± 0.004	0.176 ± 0.004	0.005	0.19	0.94
Food intake 0–8 weeks, g/day	43.2 ± 2.2 ^a^	40.4 ± 0.8 ^a^	26.6 ± 1.1 ^b^	26.0 ± 1.2 ^b^	<0.0001	0.21	0.42
Food intake 9–16 weeks, g/day	44.0 ± 1.2 ^a^	34.9 ± 0.6 ^b^	23.9 ± 0.9 ^c^	22.0 ± 1.0 ^c^	<0.0001	<0.0001	0.0009
Water intake 0–8 weeks, g/day	31.8 ± 1.6	36.8 ± 2.5	32.4 ± 1.4	33.0 ± 1.5	0.48	0.22	0.33
Water intake 9–16 weeks, g/day	21.7 ± 1.4 ^c^	36.8 ± 2.4 ^a^	28.8 ± 1.3 ^b^	37.0 ± 1.6 ^a^	0.10	<0.0001	0.12
Energy intake 0–8 weeks, kJ/day	485 ± 25 ^b^	454 ± 9 ^b^	607 ± 19 ^a^	590 ± 22 ^a^	<0.0001	0.24	0.73
Energy intake 9–16 weeks, kJ/day	470 ± 13 ^b^	392 ± 7 ^c^	536 ± 15 ^a^	533 ± 22 ^a^	<0.0001	0.0307	0.044
16-week abdominal circumference, cm	18.7 ± 0.5 ^c^	21.5 ± 0.2 ^b^	23.8 ± 0.4 ^a^	23.4 ± 0.3 ^a^	<0.0001	0.0013	<0.0001
Body mass index, g/cm^2^	0.61 ± 0.03 ^c^	0.72 ± 0.01 ^b^	0.81 ± 0.02 ^a^	0.77 ± 0.02 ^a^	<0.0001	0.09	0.0008
Retroperitoneal fat, mg/mm	210 ± 20 ^c^	256 ± 17 ^c^	619 ± 69 ^a^	488 ± 31 ^b^	<0.0001	0.24	0.018
Epididymal fat, mg/mm	89 ± 11 ^b^	90 ± 9 ^b^	199 ± 39 ^a^	182 ± 14 ^a^	<0.0001	0.67	0.63
Omental fat, mg/mm	139 ± 14 ^b^	169 ± 16 ^b^	288 ± 56 ^a^	278 ± 19 ^a^	<0.0001	0.71	0.46
Total abdominal fat, mg/mm	437 ± 42 ^c^	514 ± 37 ^c^	1107 ± 57 ^a^	948 ± 59 ^b^	<0.0001	0.47	0.042
Visceral adiposity, %	5.2 ± 0.5 ^b^	5.2 ± 0.3 ^b^	9.3 ± 1.1 ^a^	8.4 ± 0.4 ^a^	<0.0001	0.42	0.42
Liver wet weight, mg/mm	261 ± 11 ^b^	260 ± 6 ^b^	380 ± 12 ^a^	370 ± 12 ^a^	<0.0001	0.62	0.69
Cardiovascular variables							
8-week systolic blood pressure, mmHg	125 ± 4 ^b^	126 ± 2 ^b^	137 ± 3 ^a^	132 ± 2 ^a^	0.0019	0.46	0.27
16-week systolic blood pressure, mmHg	123 ± 2 ^b^	121 ± 2 ^b^	139 ± 2 ^a^	135 ± 3 ^a^	<0.0001	0.21	0.67
Left ventricle + septum, mg/mm	22.9 ± 1.1	23.8 ± 0.7	25.2 ± 1.1	24.3 ± 0.8	0.14	1.00	0.34
Right ventricle, mg/mm	4.5 ± 0.7	4.3 ± 0.3	5.3 ± 0.2	5.5 ± 0.4	0.03	1.00	0.65
Left ventricular diastolic stiffness, κ	22.1 ± 0.8 ^b^	22.4 ± 0.9 ^b^	30.1 ± 0.7 ^a^	30.2 ± 0.8 ^a^	<0.0001	0.81	0.90
Left ventricular collagen area, %	6.5 ± 0.6 ^b^	7.2 ± 0.9 ^b^	27.4 ± 2.6 ^a^	26.2 ± 2.1 ^a^	<0.0001	0.89	0.59
Metabolic variables							
Plasma triglycerides, mmol/L	0.43 ± 0.02 ^c^	0.71 ± 0.06 ^b^	1.88 ± 0.31 ^a^	1.58 ± 0.22 ^a^	<0.0001	0.96	0.15
Plasma non-esterified fatty acids, mmol/L	0.38 ± 0.06 ^b^	0.61 ± 0.06 ^a^	0.40 ± 0.03 ^b^	0.71 ± 0.07 ^a^	0.40	0.0005	0.58
Plasma total cholesterol, mmol/L	1.56 ± 0.08	1.73 ± 0.06	1.57 ± 0.10	1.71 ± 0.06	0.95	0.05	0.84
Alanine transaminase, U/L	34 ± 4	31 ± 3	38 ± 2	35 ± 3	0.25	0.39	1.00
Aspartate transaminase, U/L	116 ± 2	101 ± 6	120 ± 12	119 ± 12	0.30	0.45	0.51
Liver inflammatory cells, cells/200 µm^2^	12 ± 2 ^b^	14 ± 2 ^b^	26 ± 3 ^a^	29 ± 4 ^a^	<0.0001	0.39	0.86
Liver fat vacuoles area, fat vacuoles/200 µm^2^	21.2 ± 1.8 ^c^	22.4 ± 2.3 ^c^	135.1 ± 12.9 ^a^	75.0 ± 4.6 ^b^	<0.0001	0.0004	0.0003
Oral glucose tolerance test							
0-week basal blood glucose, mmol/L	2.6 ± 0.1	2.9 ± 0.3	2.6 ± 0.2	2.6 ± 0.2	0.58	0.58	0.58
0-week area under the curve, mmol/L·minutes	632 ± 30	598 ± 19	606 ± 19	606 ± 19	0.70	0.46	0.46
8-week basal blood glucose, mmol/L	2.9 ± 0.2	2.8 ± 0.1	3.3 ± 0.1	3.3 ± 0.1	0.001	0.7	0.68
8-week 120-min blood glucose, mmol/L	3.5 ± 0.2 ^b^	3.8 ± 0.2 ^b^	5.0 ± 0.1 ^a^	4.5 ± 0.2 ^a^	<0.0001	0.65	0.075
8-week area under the curve, mmol/L·minutes	530 ± 15 ^b^	558 ± 17 ^b^	657 ± 22 ^a^	640 ± 15 ^a^	<0.0001	0.77	0.24
16-week basal blood glucose, mmol/L	2.8 ± 0.2	2.7 ± 0.1	3.3 ± 0.2	3.0 ± 1.1	0.008	0.16	0.48
16-week 120-min blood glucose, mmol/L	3.9 ± 0.2 ^b^	4.1± 0.2 ^b^	4.8 ± 0.3 ^a^	4.8 ± 0.2 ^a^	0.002	0.68	0.68
16-week area under the curve, mmol/L·minutes	501 ± 21 ^b^	571 ± 15 ^a^	617 ± 25 ^a^	593 ± 16 ^a^	0.001	0.24	0.021
Insulin tolerance test							
8-week 120-min blood glucose, mmol/L	2.9 ± 0.4 ^b^	3.4 ± 0.4 ^b^	4.5 ± 0.3 ^a^	4.3 ± 0.2 ^a^	0.002	0.68	0.34
8-week area under the curve, mmol/L·minutes	247 ± 58 ^c^	156 ± 25 ^c^	408 ± 21 ^a^	369 ± 22 ^a^	<0.0001	0.05	0.04
16-week 120-min blood glucose, mmol/L	2.7 ± 0.3 ^b^	3.2 ± 0.3 ^b^	4.5 ± 0.4 ^a^	4.1 ± 0.2 ^a^	0.0001	0.87	0.16
16-week area under the curve, mmol/L·minutes	208 ± 37 ^c^	307 ± 36 ^b^	404 ± 54 ^a^	365 ± 16 ^a^	0.001	0.41	0.07

Values are presented as mean ± SEM, *n* = 10–12. Means in a row with unlike superscripts (a, b or c) differ, *p* < 0.05. C, rats fed with corn starch diet; CN, rats fed with corn starch diet + *Nannochloropsis oceanica*; H, rats fed with high-carbohydrate, high-fat diet; HN, rats fed with high-carbohydrate, high-fat diet + *Nannochloropsis oceanica*.

## Data Availability

The data presented in this study are available on request from the corresponding author.

## Supplementary Materials

The following are available online at https://www.mdpi.com/article/10.3390/nu13113991/s1, Table S1: PERMANOVAs based on Bray−Curtis similarity measure for square-root-transformed abundances of all rat faecal samples; Table S2: PERMANOVAs based on Euclidean distance matrix for physiological data of all rat faecal samples; Table S3: Summary of statistical tests on differential zOTU abundance; Table S4: Relative abundance of zOTUs affected by diet (ANOVA with *p* adjusted < 0.05) between C, CN, H and HN rats; Table S5: Relative abundance of zOTUs affected by treatment (ANOVA with *p* adjusted < 0.05) between C, CN, H and HN rats; Table S6: Correlation between bacterial community structure and physiological parameters (*p* < 0.05); Table S7: Taxonomic assignments of the zOTUs strongly correlated with physiological parameters; Figure S1: Effect of supplementation of diet (C or H) with *Nannochloropsis oceanica* on the ratio of Firmicutes and Bacteroidetes (F/B) abundances in rat faecal samples; Figure S2: Taxonomic profiles of bacterial communities of all faecal samples shown at the family level; Figure S3: Taxonomic profiles of bacterial communities shown at the genus level of all faecal samples.
