# Supplementary material for: Nannochloropsis oceanica as a Microalgal Food Intervention in Diet-Induced Metabolic Syndrome in Rats
Source: Nutrients. 2021 Nov 9;13(11):3991. doi: 10.3390/nu13113991 (PMC8624018; doi:10.3390/nu13113991)
Supplement: Supplementary file 1 [file nutrients-13-03991-s001.zip › nutrients-1395676-supplementary.pdf]

Supplementary file

Table S1. PERMANOVAs based on Bray-Curtis similarity measure for square-root transformed abundances of all rat faecal samples.

**PERMANOVA**						
**Source**	**df**	**SS**	**MS**	**Pseudo-F**	***P*(perm)**	**Unique perms**
Diet	1	10889	10889	11.169	0.0001	9898
Treatment	1	2598.8	2598.8	2.6657	0.0002	9868
Diet × treatment	1	1794.5	1794.5	1.8407	0.0011	9873
Res	20	19498	974.9			
Total	23	34780				
**PAIR-WISE TESTS**						
		**Source**		***t***	***P*(perm)**	**Unique perms**
		C, CN		1.7098	0.0017	461
		C, H		2.4159	0.0034	462
		C, HN		2.6766	0.0026	461
		CN, H		2.5863	0.0023	462
		CN, HN		2.7095	0.0023	462
		H, HN		1.2744	0.0209	461

P values were calculated using 9,999 permutations under a residual model. C, rats fed with corn starch diet; CN, rats fed with corn starch diet + *Nannochloropsis oceanica*; H, rats fed with high-carbohydrate, high-fat diet; HN, rats fed with high-carbohydrate, high-fat diet + *Nannochloropsis oceanica*.

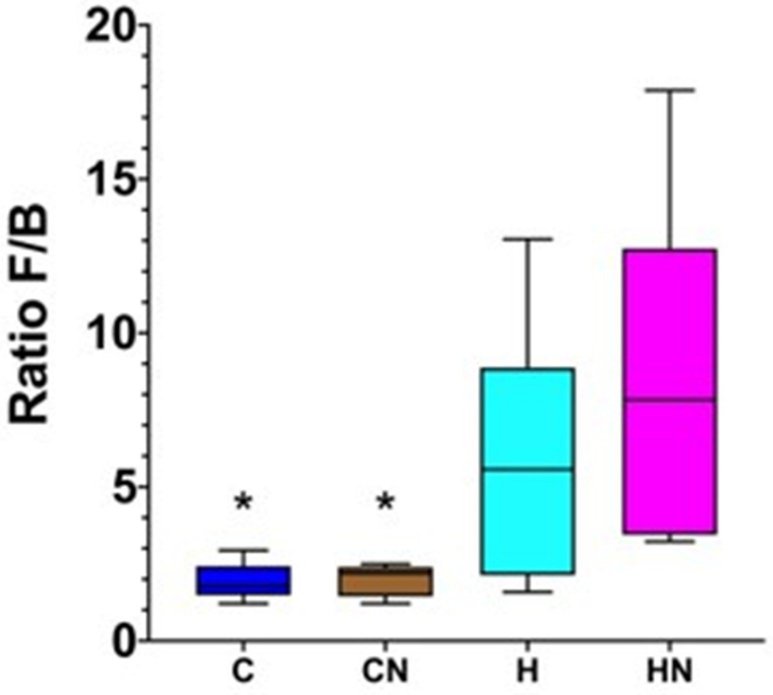

Figure S1. Effect of supplementation of diet (C or H) with *Nannochloropsis oceanica* on the ratio of Firmicutes and Bacteroidetes (F/B) abundances in rat faecal samples. Statistical analysis performed using ANOVA with Tukey's *post hoc* test for multiple comparisons, *$P<0.05$. C, rats fed with corn starch diet; CN, rats fed with corn starch diet + *Nannochloropsis oceanica*; H, rats fed with high-carbohydrate, high-fat diet; HN, rats fed with high-carbohydrate, high-fat diet + *Nannochloropsis oceanica*.

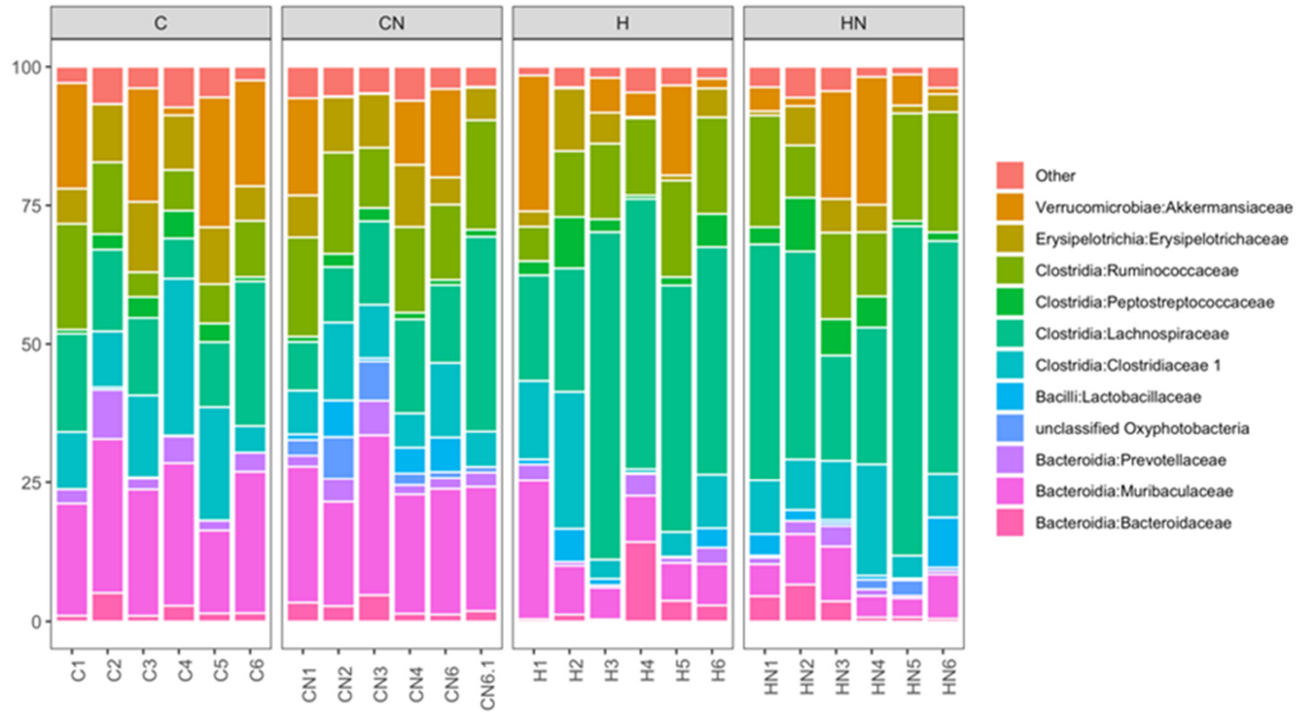

Figure S2. Taxonomic profiles of bacterial communities of all faecal samples shown at the family level. C, rats fed with corn starch diet; CN, rats fed with corn starch diet + *Nannochloropsis oceanica*; H, rats fed with high-carbohydrate, high-fat diet; HN, rats fed with high-carbohydrate, high-fat diet + *Nannochloropsis oceanica*.

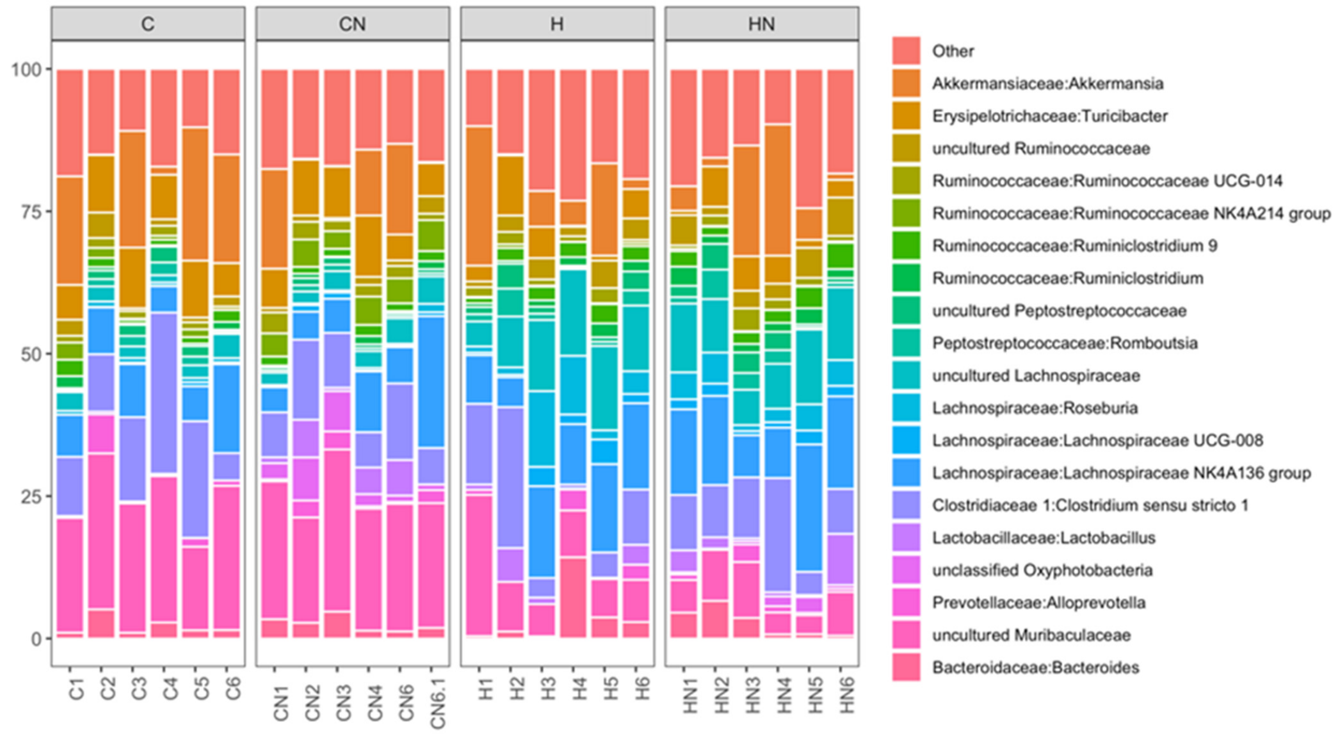

Figure S3. Taxonomic profiles of bacterial communities shown at the genus level of all faecal samples. C, rats fed with corn starch diet; CN, rats fed with corn starch diet + *Nannochloropsis oceanica*; H, rats fed with high-carbohydrate, high-fat diet; HN, rats fed with high-carbohydrate, high-fat diet + *Nannochloropsis oceanica*.

Table S2. PERMANOVAs based on Euclidean distance matrix for physiological data of all rat faecal samples.

**PERMANOVA**						
**Source**	**df**	**SS**	**MS**	**Pseudo-F**	***P*(perm)**	**Unique perms**
Diet	1	2770900	2770900	38.384	0.0001	9929
Treatment	1	218800	218800	3.031	0.0692	9944
Diet × treatment	1	1070800	1070800	14.834	0.0003	9929
Res	20	1443800	1443800			
Total	23	5504200				
**PAIR-WISE TESTS**						
	**Source**			***t***	***P*(perm)**	**Unique perms**
	C, CN			5.6852	0.0022	461
	C, H			5.5015	0.0029	462
	C, HN			7.9344	0.0018	462
	CN, H			3.0564	0.0079	462
	CN, HN			1.5282	0.16	462
	H, HN			3.1231	0.0072	462

P values were calculated using 9,999 permutations under a residual model. C, rats fed with corn starch diet; CN, rats fed with corn starch diet + *Nannochloropsis oceanica;* H, rats fed with high-carbohydrate, high-fat diet; HN, rats fed with high-carbohydrate, high-fat diet + *Nannochloropsis oceanica*.

Table S3. Summary of statistical tests on differential zOTU abundance.

**Global test (GLMs) by mvabund**		
Diet	*P*<0.0001	
Treatment	*P* = 0.002	
Diet × Treatment	*P* = 0.006	
**Univariate analysis by mvabund (*P* < 0.05)**		
**Factor**	**Number of differentially abundant OTUs**	**% of total number of OTUs**
Diet	102	7.91%
Treatment	5	0.39%
Total (unique zOTUs affected by one or more factors)	107	8.30%

Table S4. Relative abundance of zOTUs affected by diet (ANOVA with *P* adjusted <0.05) between C, CN, H and HN rats.

OTU_ID	C (%)	CN (%)	H (%)	HN (%)	Phylum	Family	Genus
Zotu35	0.63	0.50	0.00	0.01	Actinobacteria	*Bifidobacteriaceae*	*Bifidobacterium*
Zotu93	0.29	0.22	0.00	0.00	Actinobacteria	*Bifidobacteriaceae*	*Bifidobacterium*
Zotu403	0.04	0.03	0.01	0.00	Actinobacteria	unclassified	unclassified
Zotu21	0.95	0.89	0.02	0.14	Bacteroidetes	*Bacteroidaceae*	*Bacteroides*
Zotu4	7.56	7.60	1.69	1.27	Bacteroidetes	*Muribaculaceae*	unclassified
Zotu18	1.01	1.26	0.04	0.04	Bacteroidetes	*Muribaculaceae*	unclassified
Zotu20	1.11	1.21	0.16	0.09	Bacteroidetes	*Muribaculaceae*	unclassified
Zotu55	0.37	0.43	0.02	0.02	Bacteroidetes	*Muribaculaceae*	unclassified
Zotu101	0.01	0.03	0.24	0.20	Bacteroidetes	*Muribaculaceae*	unclassified
Zotu163	0.18	0.10	0.02	0.01	Bacteroidetes	*Muribaculaceae*	unclassified
Zotu401	0.27	0.30	0.06	0.04	Bacteroidetes	*Muribaculaceae*	unclassified
Zotu446	0.02	0.06	0.01	0.01	Bacteroidetes	*Muribaculaceae*	unclassified
Zotu754	0.18	0.18	0.01	0.01	Bacteroidetes	*Muribaculaceae*	unclassified
Zotu780	0.14	0.17	0.01	0.00	Bacteroidetes	*Muribaculaceae*	unclassified
Zotu812	0.12	0.12	0.01	0.01	Bacteroidetes	*Muribaculaceae*	unclassified
Zotu875	0.08	0.04	0.01	0.00	Bacteroidetes	*Muribaculaceae*	unclassified
Zotu1022	0.01	0.02	0.00	0.00	Bacteroidetes	*Muribaculaceae*	unclassified
Zotu772	0.02	0.02	0.00	0.00	Firmicutes	*Christensenellaceae*	*Christensenellaceae R-7 group*
Zotu6	4.06	1.54	0.07	0.03	Firmicutes	*Clostridiaceae 1*	*Clostridium sensu stricto 1*
Zotu615	0.02	0.02	0.00	0.00	Firmicutes	*Clostridiales vadinBB60 group*	unclassified
Zotu1143	0.00	0.00	0.01	0.01	Firmicutes	*Lachnospiraceae*	*A2*
Zotu676	0.00	0.00	0.02	0.03	Firmicutes	*Lachnospiraceae*	*Acetatifactor*
Zotu815	0.00	0.00	0.03	0.03	Firmicutes	*Lachnospiraceae*	*Acetitomaculum*
Zotu115	0.04	0.05	0.30	0.30	Firmicutes	*Lachnospiraceae*	*Anaerostipes*
Zotu237	0.01	0.00	0.07	0.11	Firmicutes	*Lachnospiraceae*	*Blautia*
Zotu91	0.07	0.05	0.15	0.42	Firmicutes	*Lachnospiraceae*	*GCA-900066575*
Zotu236	0.02	0.01	0.09	0.11	Firmicutes	*Lachnospiraceae*	*GCA-900066575*
Zotu313	0.01	0.00	0.09	0.13	Firmicutes	*Lachnospiraceae*	*GCA-900066575*
Zotu1105	0.01	0.02	0.07	0.15	Firmicutes	*Lachnospiraceae*	*GCA-900066575*
Zotu36	0.07	0.11	0.67	1.12	Firmicutes	*Lachnospiraceae*	*Lachnoclostridium*
Zotu263	0.00	0.00	0.11	0.11	Firmicutes	*Lachnospiraceae*	*Lachnoclostridium*
Zotu675	0.00	0.00	0.02	0.03	Firmicutes	*Lachnospiraceae*	*Lachnospiraceae FCS020 group*
Zotu842	0.00	0.00	0.03	0.03	Firmicutes	*Lachnospiraceae*	*Lachnospiraceae FCS020 group*
Zotu869	0.00	0.00	0.03	0.02	Firmicutes	*Lachnospiraceae*	*Lachnospiraceae FCS020 group*
Zotu50	0.01	0.03	0.81	0.51	Firmicutes	*Lachnospiraceae*	*Lachnospiraceae NK4A136 group*
Zotu64	0.11	0.78	0.02	0.02	Firmicutes	*Lachnospiraceae*	*Lachnospiraceae NK4A136 group*
Zotu137	0.00	0.00	0.20	0.35	Firmicutes	*Lachnospiraceae*	*Lachnospiraceae NK4A136 group*
Zotu195	0.01	0.01	0.19	0.20	Firmicutes	*Lachnospiraceae*	*Lachnospiraceae NK4A136 group*
Zotu253	0.00	0.00	0.19	0.08	Firmicutes	*Lachnospiraceae*	*Lachnospiraceae NK4A136 group*

Zotu286	0.00	0.00	0.07	0.19	Firmicutes	*Lachnospiraceae*	*Lachnospiraceae NK4A136 group*
Zotu290	0.01	0.00	0.19	0.02	Firmicutes	*Lachnospiraceae*	*Lachnospiraceae NK4A136 group*
Zotu297	0.09	0.22	0.00	0.00	Firmicutes	*Lachnospiraceae*	*Lachnospiraceae NK4A136 group*
Zotu328	0.00	0.00	0.10	0.08	Firmicutes	*Lachnospiraceae*	*Lachnospiraceae NK4A136 group*
Zotu353	0.00	0.00	0.07	0.07	Firmicutes	*Lachnospiraceae*	*Lachnospiraceae NK4A136 group*
Zotu398	0.00	0.00	0.06	0.09	Firmicutes	*Lachnospiraceae*	*Lachnospiraceae NK4A136 group*
Zotu422	0.00	0.00	0.07	0.06	Firmicutes	*Lachnospiraceae*	*Lachnospiraceae NK4A136 group*
Zotu513	0.00	0.00	0.09	0.08	Firmicutes	*Lachnospiraceae*	*Lachnospiraceae NK4A136 group*
Zotu661	0.00	0.00	0.02	0.03	Firmicutes	*Lachnospiraceae*	*Lachnospiraceae NK4A136 group*
Zotu787	0.00	0.00	0.01	0.02	Firmicutes	*Lachnospiraceae*	*Lachnospiraceae NK4A136 group*
Zotu917	0.00	0.00	0.01	0.02	Firmicutes	*Lachnospiraceae*	*Lachnospiraceae NK4A136 group*
Zotu1006	0.00	0.00	0.01	0.03	Firmicutes	*Lachnospiraceae*	*Lachnospiraceae NK4A136 group*
Zotu266	0.00	0.00	0.08	0.21	Firmicutes	*Lachnospiraceae*	*Lachnospiraceae UCG-001*
Zotu395	0.00	0.00	0.10	0.06	Firmicutes	*Lachnospiraceae*	*Lachnospiraceae UCG-001*
Zotu26	0.00	0.00	1.66	1.30	Firmicutes	*Lachnospiraceae*	*Lachnospiraceae UCG-006*
Zotu125	0.02	0.02	0.54	0.30	Firmicutes	*Lachnospiraceae*	*Lachnospiraceae UCG-008*
Zotu451	0.00	0.00	0.02	0.10	Firmicutes	*Lachnospiraceae*	*Lachnospiraceae UCG-008*
Zotu73	0.00	0.00	0.63	0.40	Firmicutes	*Lachnospiraceae*	*Roseburia*
Zotu373	0.09	0.03	0.00	0.00	Firmicutes	*Lachnospiraceae*	*Roseburia*
Zotu463	0.00	0.00	0.06	0.05	Firmicutes	*Lachnospiraceae*	*Roseburia*
Zotu1049	0.00	0.00	0.05	0.03	Firmicutes	*Lachnospiraceae*	*Roseburia*
Zotu27	0.00	0.00	1.16	0.74	Firmicutes	*Lachnospiraceae*	unclassified
Zotu83	0.02	0.01	0.24	0.40	Firmicutes	*Lachnospiraceae*	unclassified
Zotu112	0.00	0.00	0.29	0.19	Firmicutes	*Lachnospiraceae*	unclassified
Zotu192	0.00	0.00	0.24	0.10	Firmicutes	*Lachnospiraceae*	unclassified
Zotu210	0.00	0.00	0.13	0.21	Firmicutes	*Lachnospiraceae*	unclassified
Zotu222	0.02	0.01	0.09	0.15	Firmicutes	*Lachnospiraceae*	unclassified
Zotu332	0.00	0.00	0.12	0.07	Firmicutes	*Lachnospiraceae*	unclassified
Zotu348	0.00	0.01	0.10	0.05	Firmicutes	*Lachnospiraceae*	unclassified
Zotu416	0.00	0.01	0.06	0.12	Firmicutes	*Lachnospiraceae*	unclassified
Zotu522	0.01	0.00	0.04	0.06	Firmicutes	*Lachnospiraceae*	unclassified
Zotu534	0.00	0.00	0.05	0.02	Firmicutes	*Lachnospiraceae*	unclassified
Zotu737	0.00	0.00	0.04	0.02	Firmicutes	*Lachnospiraceae*	unclassified
Zotu944	0.00	0.00	0.01	0.02	Firmicutes	*Lachnospiraceae*	unclassified
Zotu972	0.00	0.00	0.01	0.03	Firmicutes	*Lachnospiraceae*	unclassified
Zotu1230	0.00	0.00	0.09	0.01	Firmicutes	*Lachnospiraceae*	unclassified
Zotu1235	0.00	0.00	0.33	0.03	Firmicutes	*Lachnospiraceae*	unclassified
Zotu366	0.00	0.00	0.05	0.09	Firmicutes	*Peptococcaceae*	unclassified
Zotu44	0.06	0.04	0.44	0.51	Firmicutes	*Peptostreptococcaceae*	unclassified
Zotu405	0.00	0.00	0.10	0.01	Firmicutes	*Ruminococcaceae*	*Butyricicoccus*
Zotu1161	0.00	0.00	0.00	0.01	Firmicutes	*Ruminococcaceae*	*Candidatus Soleaferrea*
Zotu172	0.12	0.17	0.00	0.00	Firmicutes	*Ruminococcaceae*	*Ruminiclostridium*

Zotu360	0.00	0.00	0.07	0.05	Firmicutes	*Ruminococcaceae*	*Ruminiclostridium*
Zotu610	0.00	0.00	0.03	0.03	Firmicutes	*Ruminococcaceae*	*Ruminiclostridium*
Zotu916	0.00	0.00	0.01	0.02	Firmicutes	*Ruminococcaceae*	*Ruminiclostridium*
Zotu387	0.03	0.08	0.01	0.01	Firmicutes	*Ruminococcaceae*	*Ruminiclostridium 5*
Zotu71	0.00	0.00	0.37	0.39	Firmicutes	*Ruminococcaceae*	*Ruminiclostridium 6*
Zotu66	0.00	0.01	0.51	0.31	Firmicutes	*Ruminococcaceae*	*Ruminiclostridium 9*
Zotu126	0.00	0.01	0.17	0.23	Firmicutes	*Ruminococcaceae*	*Ruminiclostridium 9*
Zotu196	0.00	0.00	0.15	0.10	Firmicutes	*Ruminococcaceae*	*Ruminiclostridium 9*
Zotu590	0.00	0.00	0.03	0.04	Firmicutes	*Ruminococcaceae*	*Ruminiclostridium 9*
Zotu17	0.93	2.81	0.00	0.00	Firmicutes	*Ruminococcaceae*	*Ruminococcaceae NK4A214 group*
Zotu539	0.00	0.00	0.03	0.03	Firmicutes	*Ruminococcaceae*	*Ruminococcaceae NK4A214 group*
Zotu609	0.00	0.00	0.02	0.03	Firmicutes	*Ruminococcaceae*	*Ruminococcaceae UCG-010*
Zotu843	0.00	0.00	0.01	0.02	Firmicutes	*Ruminococcaceae*	*Ruminococcaceae UCG-013*
Zotu827	0.00	0.00	0.02	0.02	Firmicutes	*Ruminococcaceae*	*Ruminococcaceae UCG-014*
Zotu76	0.23	0.41	0.01	0.00	Firmicutes	*Ruminococcaceae*	*Ruminococcus 1*
Zotu194	0.13	0.11	0.00	0.00	Firmicutes	*Ruminococcaceae*	*Ruminococcus 1*
Zotu262	0.05	0.09	0.00	0.00	Firmicutes	*Ruminococcaceae*	*Ruminococcus 2*
Zotu68	0.01	0.04	0.44	0.26	Firmicutes	*Ruminococcaceae*	unclassified
Zotu224	0.00	0.00	0.06	0.14	Firmicutes	*Ruminococcaceae*	unclassified
Zotu299	0.01	0.02	0.06	0.08	Firmicutes	*Ruminococcaceae*	unclassified
Zotu204	0.00	0.00	0.08	0.11	Tenericutes	*Mollicutes RF39*	unclassified

Differential abundance analysis was performed using Mvabund. C, rats fed with corn starch diet; CN, rats fed with corn starch diet + *Nannochloropsis oceanica*; H, rats fed with high-carbohydrate, high-fat diet; HN, rats fed with high-carbohydrate, high-fat diet + *Nannochloropsis oceanica*.

Table S5. Relative abundance of zOTUs affected by treatment (ANOVA with *P* adjusted <0.05) between C, CN, H and HN rats.

OTU_ID	C (%)	CN (%)	H (%)	HN (%)	Phylum	Family	Genus
Zotu11	0.00	3.70	0.00	0.90	Cyanobacteria	*Chloroplast*	*Nannochloropsis oceanica*
Zotu908	0.00	0.01	0.00	0.03	Firmicutes	*Ruminococcaceae*	*Butyricicoccus*
Zotu32	0.00	1.47	0.00	0.01	Firmicutes	*Ruminococcaceae*	*Ruminococcaceae NK4A214 group*
Zotu189	0.00	0.01	0.00	0.26	Firmicutes	*Ruminococcaceae*	*Ruminococcus 1*
Zotu428	0.00	0.01	0.01	0.08	Firmicutes	*Ruminococcaceae*	unclassified

Differential abundance analysis was performed using Mvabund. C, rats fed with corn starch diet; CN, rats fed with corn starch diet + *Nannochloropsis oceanica*; H, rats fed with high-carbohydrate, high-fat diet; HN, rats fed with high-carbohydrate, high-fat diet + *Nannochloropsis oceanica*.

Table S6. Correlation between bacterial community structure and physiological parameters (*P*<0.05).

Physiological variables	R^2	*P* value
Systolic blood pressure	0.86	0.001
Liver wet weight	0.81	0.001
Retroperitoneal fat	0.81	0.001
Epididymal fat	0.70	0.001
Omental fat	0.59	0.001
Feed efficiency	0.57	0.001
Kidneys wet weight	0.54	0.001
Triglycerides	0.51	0.001
Fat mass	0.47	0.003
Total abdominal fat	0.44	0.002
Oral glucose tolerance 120-minute blood glucose	0.40	0.006
Left ventricular wet weight	0.47	0.006

Table S7. Taxonomic assignments of the zOTUs strongly correlated with physiological parameters.

OTU_ID	Phylum	Family	Genus	Correlation with physiological parameters
Zotu35	Actinobacteria	*Bifidobacteriaceae*	*Bifidobacterium*	Epididymal fat (-), fat mass (-), kidneys wet weight (-), liver wet weight (-), left ventricular wet weight (-), oral glucose tolerance area under the curve (-), oral glucose tolerance 120-minute blood glucose (-), omental fat (-), retroperitoneal fat (-), systolic blood pressure (-), total abdominal fat (-), triglycerides (-), water intake (-)
Zotu93	Actinobacteria	*Bifidobacteriaceae*	*Bifidobacterium*	Liver wet weight (-)
Zotu403	Actinobacteria	unclassified	unclassified	Epididymal fat (-), fat mass (-), kidneys wet weight (-), liver wet weight (-), left ventricular wet weight (-), oral glucose tolerance area under the curve (-), oral glucose tolerance 120-minute blood glucose (-), omental fat (-), retroperitoneal fat (-), systolic blood pressure (-), total abdominal fat (-), triglycerides (-)
Zotu21	Bacteroidetes	*Bacteroidaceae*	*Bacteroides*	Alanine transaminase (+), epididymal fat (-), fat mass (-), kidneys wet weight (-), liver wet weight (-), oral glucose tolerance 120-minute blood glucose (-), omental fat (-), retroperitoneal fat (-), systolic blood pressure (-), total abdominal fat (-), triglycerides (-)
Zotu163	Bacteroidetes	*Muribaculaceae*	unclassified	Body weight (-), epididymal fat (-), fat mass (-), kidneys wet weight (-), liver wet weight (-), oral glucose tolerance 120-minute blood glucose (-), omental fat (-), retroperitoneal fat (-), systolic blood pressure (-), total abdominal fat (-), triglycerides (-)
Zotu18	Bacteroidetes	*Muribaculaceae*	unclassified	Epididymal fat (-), fat mass (-), kidneys wet weight (-), liver wet weight (-), omental fat (-), retroperitoneal fat (-), systolic blood pressure (-), total abdominal fat (-), triglycerides (-)
Zotu20	Bacteroidetes	*Muribaculaceae*	unclassified	Epididymal fat (-), fat mass (-), kidneys wet weight (-), liver wet weight (-), omental fat (-), retroperitoneal fat (-), systolic blood pressure (-), total abdominal fat (-), triglycerides (-)
Zotu4	Bacteroidetes	*Muribaculaceae*	unclassified	Epididymal fat (-), fat mass (-), kidneys wet weight (-), liver wet weight (-), omental fat (-), retroperitoneal fat (-), systolic blood pressure (-), total abdominal fat (-), triglycerides (-)
Zotu55	Bacteroidetes	*Muribaculaceae*	unclassified	Epididymal fat (-), fat mass (-), kidneys wet weight (-), liver wet weight (-), omental fat (-), retroperitoneal fat (-), systolic blood pressure (-), total abdominal fat (-), triglycerides (-)
Zotu754	Bacteroidetes	*Muribaculaceae*	unclassified	Epididymal fat (-), fat mass (-), kidneys wet weight (-), liver wet weight (-), omental fat (-), retroperitoneal fat (-), systolic blood pressure (-), total abdominal fat (-), triglycerides (-)
Zotu401	Bacteroidetes	*Muribaculaceae*	unclassified	Epididymal fat) (-), fat mass (-), liver wet weight (-), omental fat (-), retroperitoneal fat (-), systolic blood pressure (-), total abdominal fat (-)
Zotu780	Bacteroidetes	*Muribaculaceae*	unclassified	Epididymal fat (-), fat mass (-), liver wet weight (-), omental fat (-), retroperitoneal fat (-), systolic blood pressure (-), total abdominal fat (-)
Zotu812	Bacteroidetes	*Muribaculaceae*	unclassified	Epididymal fat (-), fat mass (-), liver wet weight (-), omental fat (-), retroperitoneal fat (-), systolic blood pressure (-), total abdominal fat (-)
Zotu875	Bacteroidetes	*Muribaculaceae*	unclassified	Alanine transaminase (+), epididymal fat (-), kidneys wet weight (-), liver wet weight (-), omental fat (-), retroperitoneal fat (-), total abdominal fat (-)
Zotu1022	Bacteroidetes	*Muribaculaceae*	unclassified	Epididymal fat (-), liver wet weight (-), omental fat (-), retroperitoneal fat (-), systolic blood pressure (-)
Zotu446	Bacteroidetes	*Muribaculaceae*	unclassified	Systolic blood pressure (-)
Zotu11	Cyanobacteria	*Nannochloropsis oceanica*	unclassified	Spleen wet weight (+)
Zotu772	Firmicutes	*Christensenellaceae*	*Christensenellaceae R-7 group*	Epididymal fat (-), liver wet weight (-), oral glucose tolerance area under the curve (-), oral glucose tolerance 120-minute blood glucose (-), retroperitoneal fat (-), systolic blood pressure (-)

Zotu6	Firmicutes	*Clostridiaceae 1*	*Clostridium sensu stricto 1*	Body weight (-), epididymal fat (-), fat mass (-), kidneys wet weight (-), liver wet weight (-), oral glucose tolerance area under the curve (-), oral glucose tolerance 120-minute blood glucose (-), omental fat (-), retroperitoneal fat (-), systolic blood pressure (-), total abdominal fat (-), triglycerides (-)
Zotu615	Firmicutes	*Clostridiales vadinBB60 group*	unclassified	Epididymal fat (-), fat mass (-), feed efficiency (+), liver wet weight (-), retroperitoneal fat (-)
Zotu1143	Firmicutes	*Lachnospiraceae*	*A2*	Epididymal fat (+), fat mass (+), lean mass (-), liver wet weight (+), left ventricular wet weight (+), systolic blood pressure (+)
Zotu676	Firmicutes	*Lachnospiraceae*	*Acetatifactor*	Kidneys wet weight (+), liver wet weight (+), oral glucose tolerance area under the curve (+), triglycerides (+)
Zotu815	Firmicutes	*Lachnospiraceae*	*Acetitomaculum*	Epididymal fat (+), feed efficiency (-), kidneys wet weight (+), liver wet weight (+), oral glucose tolerance 120-minute blood glucose (+), omental fat (+), retroperitoneal fat (+), systolic blood pressure (+), total abdominal fat (+), triglycerides (+)
Zotu115	Firmicutes	*Lachnospiraceae*	*Anaerostipes*	Liver wet weight (+), omental fat (+), retroperitoneal fat (+), systolic blood pressure (+), triglycerides (+)
Zotu237	Firmicutes	*Lachnospiraceae*	*Blautia*	Epididymal fat (+), fat mass (+), kidneys wet weight (+), liver wet weight (+), omental fat (+), retroperitoneal fat (+), systolic blood pressure (+), total abdominal fat (+), triglycerides (+)
Zotu236	Firmicutes	*Lachnospiraceae*	*GCA-900066575*	Epididymal fat (+), feed efficiency change (-), kidneys wet weight (+), liver wet weight (+), oral glucose tolerance 120-minute blood glucose (+), omental fat (+), retroperitoneal fat (+), systolic blood pressure (+), total abdominal fat (+), triglycerides (+)
Zotu1105	Firmicutes	*Lachnospiraceae*	*GCA-900066575*	Epididymal fat (+), kidneys wet weight (+), liver wet weight (+), omental fat (+), retroperitoneal fat (+), systolic blood pressure (+), triglycerides (+)
Zotu91	Firmicutes	*Lachnospiraceae*	*GCA-900066575*	Epididymal fat (+), kidneys wet weight (+), liver wet weight (+), omental fat (+)
Zotu313	Firmicutes	*Lachnospiraceae*	*GCA-900066575*	Kidneys wet weight (+)
Zotu263	Firmicutes	*Lachnospiraceae*	*Lachnoclostridium*	Epididymal fat (+), liver wet weight (+), oral glucose tolerance 120-minute blood glucose (+), omental fat (+), retroperitoneal fat (+)
Zotu842	Firmicutes	*Lachnospiraceae*	*Lachnospiraceae FCS020 group*	Epididymal fat (+), fat mass (+), feed efficiency (-), kidneys wet weight (+), liver wet weight (+), left ventricular wet weight (+), oral glucose tolerance area under the curve (+), oral glucose tolerance 120-minute blood glucose (+), omental fat (+), retroperitoneal fat (+), systolic blood pressure (+), total abdominal fat (+), triglycerides (+)
Zotu675	Firmicutes	*Lachnospiraceae*	*Lachnospiraceae FCS020 group*	Epididymal fat (+), fat mass (+), feed efficiency (-), kidneys wet weight (+), liver wet weight (+), left ventricular wet weight (+), oral glucose tolerance 120-minute blood glucose (+), omental fat (+), retroperitoneal fat (+), systolic blood pressure (+), total abdominal fat (+), triglycerides (+)
Zotu869	Firmicutes	*Lachnospiraceae*	*Lachnospiraceae FCS020 group*	Epididymal fat (+), feed efficiency (-), liver wet weight (+), oral glucose tolerance 120-minute blood glucose (+), omental fat (+), retroperitoneal fat (+), systolic blood pressure (+), total abdominal fat (+), triglycerides (+)
Zotu422	Firmicutes	*Lachnospiraceae*	*Lachnospiraceae NK4A136 group*	Epididymal fat (+), fat mass (+), kidneys wet weight (+), liver wet weight (+), left ventricular wet weight (+), oral glucose tolerance 120-minute blood glucose (+), omental fat (+), retroperitoneal fat (+), systolic blood pressure (+), total abdominal fat (+), triglycerides (+)
Zotu328	Firmicutes	*Lachnospiraceae*	*Lachnospiraceae NK4A136 group*	Body weight (+), epididymal fat (+), fat mass (+), liver wet weight (+), left ventricular wet weight (+), non-esterified fatty acids (+), omental fat (+), retroperitoneal fat (+), systolic blood pressure (+), total abdominal fat (+)

Zotu50	Firmicutes	*Lachnospiraceae*	*Lachnospiraceae NK4A136 group*	Epididymal fat (+), fat mass change (+), left ventricular wet weight (+), non-esterified fatty acids (+), omental fat (+), retroperitoneal fat (+), systolic blood pressure (+), total abdominal fat (+)
Zotu513	Firmicutes	*Lachnospiraceae*	*Lachnospiraceae NK4A136 group*	Epididymal fat (+), fat mass (+), left ventricular wet weight (+), non-esterified fatty acids (+), omental fat (+), retroperitoneal fat (+), systolic blood pressure (+), total abdominal fat (+)
Zotu398	Firmicutes	*Lachnospiraceae*	*Lachnospiraceae NK4A136 group*	Epididymal fat (+), kidneys wet weight (+), liver wet weight (+), omental fat (+), retroperitoneal fat (+), systolic blood pressure (+), total abdominal fat (+)
Zotu253	Firmicutes	*Lachnospiraceae*	*Lachnospiraceae NK4A136 group*	Epididymal fat (+), liver wet weight (+), oral glucose tolerance 120-minute blood glucose (+), omental fat (+), retroperitoneal fat (+), total abdominal fat (+)
Zotu917	Firmicutes	*Lachnospiraceae*	*Lachnospiraceae NK4A136 group*	Kidneys wet weight (+), liver wet weight (+), omental fat (+), retroperitoneal fat (+), systolic blood pressure (+)
Zotu290	Firmicutes	*Lachnospiraceae*	*Lachnospiraceae NK4A136 group*	Non-esterified fatty acids (+), omental fat (+), retroperitoneal fat (+)
Zotu286	Firmicutes	*Lachnospiraceae*	*Lachnospiraceae NK4A136 group*	Liver wet weight (+), triglycerides (+)
Zotu787	Firmicutes	*Lachnospiraceae*	*Lachnospiraceae NK4A136 group*	Systolic blood pressure (+), triglycerides (+)
Zotu137	Firmicutes	*Lachnospiraceae*	*Lachnospiraceae NK4A136 group*	Systolic blood pressure (+)
Zotu195	Firmicutes	*Lachnospiraceae*	*Lachnospiraceae NK4A136 group*	Systolic blood pressure (+)
Zotu297	Firmicutes	*Lachnospiraceae*	*Lachnospiraceae NK4A136 group*	Feed efficiency (+)
Zotu353	Firmicutes	*Lachnospiraceae*	*Lachnospiraceae NK4A136 group*	Systolic blood pressure (+)
Zotu64	Firmicutes	*Lachnospiraceae*	*Lachnospiraceae NK4A136 group*	Feed efficiency (+)
Zotu395	Firmicutes	*Lachnospiraceae*	*Lachnospiraceae UCG-001*	Epididymal fat (+), liver wet weight (+), oral glucose tolerance 120-minute blood glucose (+), omental fat (+), retroperitoneal fat (+), systolic blood pressure (+), total abdominal fat (+), triglycerides (+)

Zotu266	Firmicutes	*Lachnospiraceae*	*Lachnospiraceae UCG-001*	Epididymal fat (+), kidneys wet weight (+), liver wet weight (+), systolic blood pressure (+), triglycerides (+)
Zotu26	Firmicutes	*Lachnospiraceae*	*Lachnospiraceae UCG-006*	Epididymal fat (+), fat mass (+), kidneys wet weight (+), liver wet weight (+), left ventricular wet weight (+), oral glucose tolerance 120-minute blood glucose (+), omental fat (+), retroperitoneal fat (+), systolic blood pressure (+), total abdominal fat (+), triglycerides (+)
Zotu125	Firmicutes	*Lachnospiraceae*	*Lachnospiraceae UCG-008*	Epididymal fat (+), non-esterified fatty acids (+), omental fat (+), retroperitoneal fat (+), systolic blood pressure (+)
Zotu451	Firmicutes	*Lachnospiraceae*	*Lachnospiraceae UCG-008*	Kidneys wet weight (+), triglycerides (+)
Zotu1049	Firmicutes	*Lachnospiraceae*	*Roseburia*	Epididymal fat (+), fat mass (+), liver wet weight (+), left ventricular wet weight (+), non-esterified fatty acids (+), omental fat (+), retroperitoneal fat (+), systolic blood pressure (+), total abdominal fat (+)
Zotu373	Firmicutes	*Lachnospiraceae*	*Roseburia*	Body weight (-), epididymal fat (-), kidneys wet weight (-), liver wet weight (-), retroperitoneal fat (-), total abdominal fat (-)
Zotu73	Firmicutes	*Lachnospiraceae*	*Roseburia*	Epididymal fat (+), fat mass (+), left ventricular wet weight (+), non-esterified fatty acids (+), omental fat (+)
Zotu463	Firmicutes	*Lachnospiraceae*	*Roseburia*	Retroperitoneal fat (+), right ventricular wet weight (+), systolic blood pressure (+)
Zotu737	Firmicutes	*Lachnospiraceae*	unclassified	Epididymal fat (+), fat mass (+), liver wet weight (+), left ventricular wet weight (+), non-esterified fatty acids (+), oral glucose tolerance 120-minute blood glucose (+), omental fat (+), retroperitoneal fat (+), systolic blood pressure (+), total abdominal fat (+)
Zotu1230	Firmicutes	*Lachnospiraceae*	unclassified	Epididymal fat (+), fat mass change (+), liver wet weight (+), left ventricular wet weight (+), oral glucose tolerance 120-minute blood glucose (+), omental fat (+), retroperitoneal fat (+), systolic blood pressure (+), total abdominal fat (+)
Zotu73	Firmicutes	*Lachnospiraceae*	*Roseburia*	Epididymal fat (+), fat mass (+), left ventricular wet weight (+), non-esterified fatty acids (+), omental fat (+)
Zotu463	Firmicutes	*Lachnospiraceae*	*Roseburia*	Retroperitoneal fat (+), right ventricular wet weight (+), systolic blood pressure (+)
Zotu737	Firmicutes	*Lachnospiraceae*	unclassified	Epididymal fat (+), fat mass change (+), liver wet weight (+), left ventricular wet weight (+), non-esterified fatty acids (+), oral glucose tolerance 120-minute blood glucose (+), omental fat (+), retroperitoneal fat (+), systolic blood pressure (+), total abdominal fat (+)
Zotu1230	Firmicutes	*Lachnospiraceae*	unclassified	Epididymal fat (+), fat mass (+), liver wet weight (+), left ventricular wet weight (+), oral glucose tolerance 120-minute blood glucose (+), omental fat (+), retroperitoneal fat (+), systolic blood pressure (+), total abdominal fat (+)
Zotu192	Firmicutes	*Lachnospiraceae*	unclassified	Epididymal fat (+), fat mass (+), liver wet weight (+), non-esterified fatty acids (+), oral glucose tolerance 120-minute blood glucose (+), omental fat (+), retroperitoneal fat (+), systolic blood pressure (+), total abdominal fat (+)
Zotu332	Firmicutes	*Lachnospiraceae*	unclassified	Epididymal fat (+), fat mass (+), liver wet weight (+), left ventricular wet weight (+), oral glucose tolerance 120-minute blood glucose (+), omental fat (+), retroperitoneal fat (+), systolic blood pressure (+), total abdominal fat (+)
Zotu522	Firmicutes	*Lachnospiraceae*	unclassified	Epididymal fat (+), feed efficiency (-), kidneys wet weight (+), liver wet weight (+), omental fat (+), retroperitoneal fat (+), systolic blood pressure (+), total abdominal fat (+), triglycerides (+)
Zotu112	Firmicutes	*Lachnospiraceae*	unclassified	Epididymal fat (+), feed efficiency (-), liver wet weight (+), oral glucose tolerance 120-minute blood glucose (+), omental fat (+), retroperitoneal fat (+), systolic blood pressure (+), total abdominal fat (+)
Zotu27	Firmicutes	*Lachnospiraceae*	unclassified	Epididymal fat (+), feed efficiency (-), liver wet weight (+), oral glucose tolerance 120-minute blood glucose (+), omental fat (+), retroperitoneal fat (+), systolic blood pressure (+), total abdominal fat (+)

Zotu83	Firmicutes	*Lachnospiraceae*	unclassified	Epididymal fat (+), fat mass (+), kidneys wet weight (+), liver wet weight (+), omental fat (+), retroperitoneal fat (+), systolic blood pressure (+), triglycerides (+)
Zotu944	Firmicutes	*Lachnospiraceae*	unclassified	Epididymal fat (+), fat mass (+), kidneys wet weight (+), liver wet weight (+), omental fat (+), retroperitoneal fat (+), systolic blood pressure (+), total abdominal fat (+)
Zotu416	Firmicutes	*Lachnospiraceae*	unclassified	Epididymal fat (+), kidneys wet weight (+), liver wet weight (+), omental fat (+), retroperitoneal fat (+), systolic blood pressure (+), triglycerides (+)
Zotu534	Firmicutes	*Lachnospiraceae*	unclassified	Epididymal fat (+), liver wet weight (+), oral glucose tolerance 120-minute blood glucose (+), omental fat (+), retroperitoneal fat (+), systolic blood pressure (+), total abdominal fat (+)
Zotu210	Firmicutes	*Lachnospiraceae*	unclassified	Epididymal fat (+), kidneys wet weight (+), liver wet weight (+), omental fat (+), retroperitoneal fat (+), systolic blood pressure (+)
Zotu1235	Firmicutes	*Lachnospiraceae*	unclassified	Epididymal fat (+), oral glucose tolerance 120-minute blood glucose (+), omental fat (+), retroperitoneal fat (+), total abdominal fat (+)
Zotu222	Firmicutes	*Lachnospiraceae*	unclassified	Kidneys wet weight (+), liver wet weight (+), retroperitoneal fat (+), systolic blood pressure (+), triglycerides (+)
Zotu348	Firmicutes	*Lachnospiraceae*	unclassified	Retroperitoneal fat (+), systolic blood pressure (+)
Zotu366	Firmicutes	*Peptococcaceae*	unclassified	Kidneys wet weight (+), liver wet weight (+), systolic blood pressure (+), triglycerides (+)
Zotu405	Firmicutes	*Ruminococcaceae*	*Butyricicoccus*	Epididymal fat (+), oral glucose tolerance 120-minute blood glucose (+), omental fat (+), retroperitoneal fat (+), total abdominal fat (+)
Zotu908	Firmicutes	*Ruminococcaceae*	*Butyricicoccus*	Triglycerides (+)
Zotu1161	Firmicutes	*Ruminococcaceae*	*Candidatus Soleaferrea*	Right ventricular wet weight (+), triglycerides (+)
Zotu916	Firmicutes	*Ruminococcaceae*	*Ruminiclostridium*	Epididymal fat (+), kidneys wet weight (+), liver wet weight (+), omental fat (+), retroperitoneal fat (+), systolic blood pressure (+), total abdominal fat (+), triglycerides (+)
Zotu172	Firmicutes	*Ruminococcaceae*	*Ruminiclostridium*	Epididymal fat (-), feed efficiency (-), liver wet weight (-), oral glucose tolerance area under the curve (-), oral glucose tolerance 120-minute blood glucose (-), retroperitoneal fat (-), systolic blood pressure (-)
Zotu360	Firmicutes	*Ruminococcaceae*	*Ruminiclostridium*	Epididymal fat (+), omental fat (+), retroperitoneal fat (+), systolic blood pressure (+)
Zotu610	Firmicutes	*Ruminococcaceae*	*Ruminiclostridium*	Non-esterified fatty acids (+), retroperitoneal fat (+), right ventricular wet weight (+), systolic blood pressure (+)
Zotu387	Firmicutes	*Ruminococcaceae*	*Ruminiclostridium 5*	Epididymal fat (-), feed efficiency (+), liver wet weight (-), retroperitoneal fat (-), systolic blood pressure (-), triglycerides (-)
Zotu71	Firmicutes	*Ruminococcaceae*	*Ruminiclostridium 6*	Kidneys wet weight (+), retroperitoneal fat (+), systolic blood pressure (+)
Zotu66	Firmicutes	*Ruminococcaceae*	*Ruminiclostridium 9*	Epididymal fat (+), fat mass (+), liver wet weight (+), non-esterified fatty acids (+), omental fat (+), retroperitoneal fat (+), systolic blood pressure (+), total abdominal fat (+)
Zotu126	Firmicutes	*Ruminococcaceae*	*Ruminiclostridium 9*	Epididymal fat (+), kidneys wet weight (+), liver wet weight (+), omental fat (+), retroperitoneal fat (+), systolic blood pressure (+), total abdominal fat (+)
Zotu590	Firmicutes	*Ruminococcaceae*	*Ruminiclostridium 9*	Epididymal fat (+), kidneys wet weight (+), liver wet weight (+), omental fat (+), retroperitoneal fat (+), systolic blood pressure (+), triglycerides (+)
Zotu196	Firmicutes	*Ruminococcaceae*	*Ruminiclostridium 9*	Liver wet weight (+), oral glucose tolerance area under the curve (+), systolic blood pressure (+), triglycerides (+)

Zotu17	Firmicutes	*Ruminococcaceae*	*Ruminococcaceae NK4A214 group*	Epididymal fat (-), feed efficiency (+), liver wet weight (-), retroperitoneal fat (-), systolic blood pressure (-), triglycerides (-)
Zotu539	Firmicutes	*Ruminococcaceae*	*Ruminococcaceae NK4A214 group*	Epididymal fat (+), kidneys wet weight (+), liver wet weight (+), omental fat (+), retroperitoneal fat (+), systolic blood pressure (+)
Zotu32	Firmicutes	*Ruminococcaceae*	*Ruminococcaceae NK4A214 group*	Feed efficiency (+), systolic blood pressure (-)
Zotu609	Firmicutes	*Ruminococcaceae*	*Ruminococcaceae UCG-010*	Epididymal fat (+), kidneys wet weight (+), right ventricular wet weight (+), systolic blood pressure (+)
Zotu843	Firmicutes	*Ruminococcaceae*	*Ruminococcaceae UCG-013*	Liver wet weight (+)
Zotu827	Firmicutes	*Ruminococcaceae*	*Ruminococcaceae UCG-014*	Oral glucose tolerance area under the curve (+), systolic blood pressure (+), triglycerides (+)
Zotu76	Firmicutes	*Ruminococcaceae*	*Ruminococcus 1*	Epididymal fat (-), fat mass (-), feed efficiency (-), liver wet weight (-), omental fat (-), retroperitoneal fat (-), systolic blood pressure (-), triglycerides (-)
Zotu194	Firmicutes	*Ruminococcaceae*	*Ruminococcus 1*	Epididymal fat (-), fat mass (-), liver wet weight (-), retroperitoneal fat (-), systolic blood pressure (-)
Zotu189	Firmicutes	*Ruminococcaceae*	*Ruminococcus 1*	Kidneys wet weight (+), triglycerides (+)
Zotu262	Firmicutes	*Ruminococcaceae*	*Ruminococcus 2*	Systolic blood pressure (-)
Zotu68	Firmicutes	*Ruminococcaceae*	unclassified	Epididymal fat (+), fat mass (+), liver wet weight (+), non-esterified fatty acids (+), omental fat (+), retroperitoneal fat (+), systolic blood pressure (+), total abdominal fat (+)
Zotu299	Firmicutes	*Ruminococcaceae*	unclassified	Epididymal fat (+), kidneys wet weight (+), liver wet weight (+), omental fat (+), retroperitoneal fat (+), systolic blood pressure (+), triglycerides (+)
Zotu428	Firmicutes	*Ruminococcaceae*	unclassified	Kidneys wet weight (+), triglycerides (+)
Zotu224	Firmicutes	*Ruminococcaceae*	unclassified	Triglycerides (+)
Zotu204	Tenericutes	unclassified	unclassified	Epididymal fat (+), fat mass (+), kidneys wet weight (+), liver wet weight (+), omental fat (+), retroperitoneal fat (+), systolic blood pressure (+), triglycerides (+)

Differential abundance analysis was performed using Mvabund. This table includes the physiological parameters strongly correlated ($P<0.05$) with the bacterial community and incorporates zOTUs that interact with at least 1 of these parameters. Plus sign (+) indicates positive correlations, while minus sign (−) indicates negative correlation.
